# An anisotropic linear thermo-viscoelastic constitutive law: Elastic relaxation and thermal expansion creep in the time domain

**DOI:** 10.1007/s11043-017-9364-x

**Published:** 2017-09-19

**Authors:** Heinz E. Pettermann, Antonio DeSimone

**Affiliations:** 1 Institute of Lightweight Design and Structural Biomechanics, Vienna University of Technology, Vienna, Austria; 2 Scuola Internazionale Superiore di Studi Avanzati – SISSA, Trieste, Italy

**Keywords:** Viscoelastic, Anisotropic, Constitutive laws, Thermal expansion creep, Finite element method implementation

## Abstract

A constitutive material law for linear thermo-viscoelasticity in the time domain is presented. The time-dependent relaxation formulation is given for full anisotropy, i.e., both the elastic and the viscous properties are anisotropic. Thereby, each element of the relaxation tensor is described by its own and independent Prony series expansion. Exceeding common viscoelasticity, time-dependent thermal expansion relaxation/creep is treated as inherent material behavior. The pertinent equations are derived and an incremental, implicit time integration scheme is presented.

The developments are implemented into an implicit FEM software for orthotropic material symmetry under plane stress assumption. Even if this is a reduced problem, all essential features are present and allow for the entire verification and validation of the approach. Various simulations on isotropic and orthotropic problems are carried out to demonstrate the material behavior under investigation.

## Introduction

The apparent effects of viscoelastic material behavior manifest themselves in a time-dependent response to loading and accompanying energy dissipation. Relaxation or creep occurs when a viscoelastic material is exposed to quasi-static loads and load changes. Under cyclic excitation damping is exhibited. These phenomena are treated typically in the time and in the frequency domain, respectively.

Such viscoelastic effects are widespread in natural as well as in engineering materials. Among them are almost all biological tissues and most polymers, in particular thermoplastic materials. Biological materials are composites “by nature”, whereas engineering polymers are often mixed with other constituents to improve their performance. These materials are likely to exhibit thermal expansion relaxation/creep behavior, i.e. time-dependent changes of unconstrained thermal strains as a response to temperature changes. Such composites, natural and man made ones, often show direction dependent properties and the consideration of anisotropy becomes inevitable.

A general introduction to viscoelasticity can be found, e.g. in Lakes (2009), Gurtin and Sternberg (1962), Schapery (2000), which focus predominantly on isotropic behavior and treat the time as well as the frequency domain. Transversely isotropic (and orthotropic) viscoelastic models have been presented, e.g. in Holzapfel (2000), Bergström and Boyce (2001), Pandolfi and Manganiello (2006), Puso and Weiss (1998), Taylor et al. (2009), Nedjar (2007), based on invariants of the strain representation, which are commonly used for biological soft tissues. Typically, they combine non-linear orthotropic elasticity with linear isotropic viscosity. Linear viscoelasticity of orthotropic media is presented in Carcione (1995), Dong and McMechan (1995) in the context of geo-materials. Linear and non-linear orthotropic viscoelasticity adopting not the full set of orthotropic viscous effects is presented in Melo and Radford (2004), Kaliske (2000) and applied to composites and foam materials, respectively. Full elastic anisotropy is treated in Lévesque et al. (2008), but with one single relaxation function operating on the entire tensor. In Schapery (1964) full anisotropy is modeled with various relaxation functions applied to elasticity tensor contributions. A review on damping of composites is given in Chandra et al. (1999). Anisotropic non-linear viscoelastic constitutive laws have been introduced in Schapery (1969), Zocher et al. (1997), Sawant and Muliana (2008), Poon and Ahmad (1998) which found widespread application to study composite materials. Implementation aspects are discussed in Crochon et al. (2010), Sorvari and Hämäläinen (2010).

Relaxation type effects occurring for the thermal expansion behavior are mentioned in Lakes (2009), Zocher et al. (1997), Schapery (1964), and observed experimentally for polymers, e.g. Spencer and Boyer (1946). A hygro-thermal expansion relaxation function is presented in Bažant (1970) in the context of concrete behavior. Otherwise, to the knowledge of the authors, there are no modeling attempts in the literature for thermal expansion creep. It is noted that thermal expansion relaxation/creep, here, does not refer to secondary effects arising, e.g. from transient thermal fields, but solely denotes the time-dependent response as inherent material behavior.

In the present work linear thermo-viscoelasticity of anisotropic materials is treated in the time domain. Thereby the thermo-elastic as well as the viscous responses are considered to behave anisotropically. The temperature dependence for thermorheologically simple materials is accounted for by a time–temperature shift function. Time-dependent, anisotropic thermal expansion relaxation/creep is treated.

The constitutive formulations are developed and the pertinent equations are given, including the consistent material tangent operator. The state of the material is handled by internal variables. The algorithms are implemented into an implicit Finite Element Method (FEM) software package. This is done under the assumption of plane stress states and orthotropic materials, but the approach is general and the implementation can be extended to tri-axial problems and general anisotropy in a straightforward manner just by adding additional terms of the same form.

For examples of applications, homogenized orthotropic material data is computed from a simple unit cell approach which serves as input to the developed constitutive laws. This allows for a consistent verification and validation of the developments. Not only the principal load cases are simulated to extract the input data, but also general loading scenarios and loading histories.

## Linear viscoelastic constitutive model

The constitutive model for considerations in time domain has to give the stress response as a function of the strain (rate) history and the appropriate instantaneous material behavior. For isotropic material symmetry the stress and strain tensors can be split into their volumetric and deviatoric part. The corresponding scalar valued material properties are the bulk and shear relaxation functions. A similar decomposition can be performed for cubic material symmetry (e.g. used in Pettermann and Hüsing 2012) and for the transversely isotropic case; see e.g. Suquet and Bornert (2001). Alternatively, one can deal with the tensorial equations, as is pursed in the present work which allows for the treatment of the most general anisotropic case. Both the elastic and the viscous contributions to the response are allowed to be of general anisotropy.

Adopting Voigt notation, the hereditary integral in tensorial form reads

1 $$ \sigma_{i} (t) = \int_{0}^{t} R_{ij} (t-s) \dot{\varepsilon }_{j} (s)\, ds. $$

Stress and strain are given by

2 $$ \sigma_{i} = ( \sigma_{11} \; \sigma_{22} \; \sigma_{33} \; \sigma _{12} \; \sigma_{13} \; \sigma_{23} )^{T}, \qquad \varepsilon _{i} = ( \varepsilon_{11} \; \varepsilon_{22} \; \varepsilon_{33} \; \gamma_{12} \; \gamma_{13} \; \gamma_{23} )^{T}, $$

with $\gamma_{ij} = 2 \varepsilon_{ij}$. Note that in Voigt notation, coordinate transformation operators must be formulated correspondingly. The time-dependent material tensor for anisotropic materials is given by the symmetric $6\times 6$ matrix, $R_{ij}(t) = R_{ji}(t)$. In the most general case it is composed of 21 individual relaxation functions of the form,

3 $$ R_{ij}(t) = R_{ij \; 0} \biggl[ 1 - \sum _{k} r_{ij \; k} \bigl( 1 - \exp \bigl( -t / \tau^{r_{ij \; k}}\bigr) \bigr)\biggr] \quad (\mbox{no sum on}\ ij) , $$

which are given by Prony series expansions with $R_{ij \; 0}$ being the instantaneous values, i.e., the elasticity tensor elements giving the short term behavior. The sums over $k$ Prony terms contain the relative relaxation elements, $r_{ij \; k}$, and the corresponding characteristic times, $\tau^{r_{ij \; k}}$. Note that the relaxation tensor, $R_{ij}(t)$, possesses off-diagonal terms with $i,j \le 3$, implicitly containing some Poisson relaxation which is not necessarily monotonic; see e.g. Lakes and Wineman (2006). The stress components can be expressed by combining Eqs. (1) and (3) as,

4 $$ \sigma_{i}(t) = \sum_{j=1}^{6} R_{ij \; 0} \biggl[ \varepsilon_{j} - \sum _{k} \frac{r_{ij \; k} }{\tau ^{r_{ij \; k}} } \int^{t}_{0} \exp \bigl( -s / \tau^{r_{ij \; k}} \bigr) \varepsilon_{j} (t-s)\, ds \biggr] , $$

where the sum over $j$ gives the contributions of the individual strain components. The “relative creep contribution” for each Prony term,

5 $$ e_{{i}\; k}^{j} = \frac{ 1 }{\tau ^{r_{ij \; k}} } \int^{t}_{0} \exp \bigl( -s / \tau^{r_{ij \; k}} \bigr) \varepsilon_{j} (t-s)\, ds , $$

is introduced and summed to the “total creep contributions” by

6 $$ \epsilon_{i}^{j} = \sum_{k} r_{ij \; k} \; e_{{i}\; k}^{j} , $$

where the superscript indicates the cause (i.e., the applied strain) and the subscript denotes the stress component in Eq. (4) which is affected. This unusual super/subscript notation is chosen to discern different types of “strain like” quantities unambiguously. Finally, the stress components read,

7 $$ \sigma_{i} = \sum_{j=1}^{6} R_{ij \; 0} \bigl[ \varepsilon_{j} - \epsilon _{i}^{j} \bigr] . $$

Note that the expressions $\epsilon_{i}^{j}$ and $e_{i \; k}^{j}$ cannot be interpreted as creep strains, and $\epsilon_{i}^{j} \ne \epsilon_{j}^{i} $. Consequently, they are rather property type quantities carrying information on how much of the relaxation potential has been consumed until time $t$. Nevertheless, the $e_{i \; k}^{j}$ for every Prony term $k$ represent the internal state variables.

The proposed approach may be viewed as an *ad hoc* extension of common linear viscoelastic models. Its thermodynamic consistency, however, is not ensured and could be violated by a unsuited set of material parameters.

The temperature dependence of viscoelastic thermorheologically simple materials is widely treated by introducing a reduced time; see e.g. Lakes (2009),

8 $$ t_{\mathrm{red}} = \frac{ t }{A(T) } $$

with the shift function, $A(T)$, depending on the temperature, $T$. In the present work the empirical Williams–Landel–Ferry (WLF) function, see e.g. Lakes (2009),

9 $$ \log A(T) = - \frac{ C_{1} (T-T_{\mathrm{ref}}) }{C_{2} + (T-T_{\mathrm{ref}}) } , $$

is used with $T_{\mathrm{ref}}$ being the reference temperature and $C_{1}$ and $C_{2}$ are constants pertaining to the particular material under consideration. In general cases, each relaxation element, $R_{ij}(t)$, can have its own time–temperature shift function, $A_{ij}(T)$.

## Thermal expansion relaxation/creep constitutive model

In linear thermo-(visco)elasticity assuming small strains, additive decomposition can be applied, which gives the total strain (in Voigt notation),

10 $$ \varepsilon_{i}^{\mathrm{tot}} = \varepsilon_{i}^{\mathrm{ve}} + \varepsilon _{i}^{\mathrm{th}} , $$

as the sum of the viscoelastic ^ve^ and the thermal ^th^ contributions.

Now, materials may show thermal expansion which exhibits time dependence as an inherent material behavior (e.g. composites). Following Lakes (2009), Zocher et al. (1997), the time-dependent thermal expansion relaxation is given by

11 $$ \varepsilon_{i}^{\mathrm{th}} (t) = \int_{0}^{t} \alpha_{i} (t-s) \dot{ \vartheta }(s) \,ds, $$

with $\alpha_{i}$ being the time-dependent coefficients of thermal expansion and, here, temperature independent behavior is assumed. $\vartheta $ is the temperature change from some (stress free) starting temperature. For the time independent case, the usual thermal expansion results from integration of Eq. (11) as $\varepsilon_{i} ^{\mathrm{th}} = \alpha_{i} \vartheta $. Note that Eq. (11) exactly resembles the hereditary integral in Eq. (1) for the time-dependent stresses under strain loading. Thus, the further treatment goes in perfect analogy to linear viscoelasticity as studied before.

For the most general anisotropic case, the time-dependent thermal expansion tensor (in correspondence to the strain in Eq. (2)) reads

12 $$ \alpha_{i} (t) = \bigl[\; \alpha_{11} (t) \; \alpha_{22} (t) \; \alpha_{33} (t) \; \alpha_{12} (t) \; \alpha_{13} (t) \; \alpha_{23} (t)\; \bigr]^{T} . $$

The components are given by the six individual relaxation functions,

13 $$ \alpha_{i}(t) = \alpha_{i \; 0} \biggl[ 1 - \sum _{k} a_{i \; k} \bigl( 1 - \exp \bigl( -t / \tau^{a_{i \; k}}\bigr) \bigr)\biggr] \quad (\mbox{no sum on}\ i) , $$

with the instantaneous thermal expansion coefficients $\alpha_{i \; 0}$ and the relative relaxation values $a_{i \; k}$ as well as characteristic times $\tau^{a_{i \; k}}$ for the Prony series with $k$ terms. For common materials, the relative relaxation values, $a_{i \; k}$, are negative which give rise to creep type thermal expansion behavior.

Applying Eqs. (4) to (7) analogously, one obtains the thermal strain:

14 $$ \varepsilon_{i}^{\mathrm{th}} = \alpha_{i \; 0} \biggl[ \vartheta - \sum_{k} a_{i \; k} \; \theta_{i \; k} \biggr] \quad (\mbox{no sum on}\ i) . $$

The “relative creep temperatures”, $\theta_{i \; k}$, have no direct physical meaning, however, they carry the information on the amount of relaxation and, consequently, are used as internal state variables. The concept of reduced time and a shift function, Eq. (8), can be applied equivalently.

The viscoelasticity and the thermal expansion relaxation have to be solve simultaneously, fulfilling Eq. (10).

## Time integration and FEM implementation

The scheme of the implicit incremental time solution for arbitrary strain histories in the context of FEM can be found e.g. in Crochon et al. (2010), Sorvari and Hämäläinen (2010), Poon and Ahmad (1998), Muliana and Khan (2008). The pertinent equations are given briefly in the following. Key to the stress update and the consistent Jacobian formulation is the time integration of Eq. (1) with the material properties given in Eq. (3). Besides the strain rate, the only time-dependent contributions are the exponential functions in Eq. (3). They appear in all elements of $R_{ij}(t)$, just with different characteristic times. With focus on these terms one can extract an integral of the form,

15 $$ e_{i}^{j} = \int_{0}^{t} \bigl( 1 - \exp \bigl( s-t / \tau^{r_{ij \; k}}\bigr) \bigr) \frac{d \varepsilon _{j} }{d s} \,d s . $$

where ${d \varepsilon_{j} / d s}$ is the appropriate strain rate. Here, $e_{i}^{j}$ is the “relative creep contribution” as introduced in Eq. (5). The above equation is solved for a finite increment of time, $\Delta t$, added to time $t^{\mathrm{n}}$ at which the state is completely known and under the assumption that the strain increases linearly with time. This yields the increment of the relative creep contribution as,

16 $$ \Delta e_{i}^{j} = \frac{\tau ^{r_{ij \; k}} }{\Delta t} \biggl( \frac{\Delta t }{\tau ^{r_{ij \; k}}} + \exp \biggl(- \frac{\Delta t }{\tau ^{r_{ij \; k}}}\biggr) - 1 \biggr) \Delta \varepsilon_{j} + \biggl( 1 - \exp \biggl(-\frac{\Delta t }{\tau ^{r_{ij \; k}}}\biggr) \biggr) \bigl( \varepsilon_{j} ^{\; \mathrm{n}} - e_{i}^{j \; \mathrm{n}} \bigr) , $$

with the superscript ^n^ denoting the state at the beginning of the increment. With this expression the stress update can be given as

17 $$ \Delta \sigma_{i} = \sum_{j=1}^{6} R_{ij \; 0} \biggl[ \Delta \varepsilon _{j} - \sum _{k} r_{ij \; k} \Delta e_{i \; k}^{j} \biggr] . $$

The role of the solution dependent state variables is taken by $e_{i \; k}^{j}$, accumulated over the time increments. The equation for computing the consistent tangent moduli elements depends on the time increment only,

18 $$ R_{ij}^{\mathrm{{tangent}}} = R_{ij \; 0} \biggl[ 1 - \sum _{k} r_{ij \; k} \; \frac{\tau ^{r_{ij \; k}} }{\Delta t} \biggl( \frac{\Delta t }{\tau ^{r_{ij \; k}}} + \exp \biggl(- \frac{\Delta t }{\tau ^{r_{ij \; k}}}\biggr) -1 \biggr) \biggr] , $$

and does not include any matrix summation. In the implementation of this solution scheme, “symmetry” on the creep contributions $e_{i \; k}^{j}$ (and $\epsilon_{i}^{j}$) must not be enforced; see Eq. (7). However, the material input requires $R_{ij \; 0} = R_{ji \; 0}$, $r_{ij \; k} = r_{ji \; k}$, and $\tau^{r_{ij \; k}} = \tau^{r_{ji \; k}} $, to maintain symmetry in the relaxation tensor at any time.

The treatment of thermal expansion relaxation/creep is equivalent to the stress update, because of the identical structure of the governing equations, and it yields the increment of thermal strain components,

19 $$ \Delta \varepsilon_{i}^{\mathrm{th}} = \alpha_{i \; 0} \biggl[ \Delta \vartheta - \sum_{k} a_{i \; k} \; \Delta \theta_{i \; k} \biggr] , \quad (\mbox{no sum on}\ i). $$

Similar to the consistent tangent moduli, the coefficients of thermal expansion at the end of the time increment are required for effective convergence. They are computed in perfect analogy with Eq. (18).

### Orthotropic plane stress implementation

The implementation of the viscoelastic model is carried out with the UMAT option, the thermal expansion with the UEXPAN option, for the implicit FEM software package ABAQUS/Standard v6.14 *(Dassault Systèmes Simulia Corp., Providence, RI, USA)*. The present implementation allows for 13 Prony terms maximum for any of the relaxation type properties. It is carried out for the case of orthotropic material symmetry under plane stress conditions. Even if this poses a reduced problem, all essential features of the proposed constitutive law are included, and the model formulation and the software realization can be tested and verified entirely. Extensions to more general cases just need the consideration of additional terms in the summation loops of exactly the same type.

For constant temperatures which are not equal to the WLF-reference temperature, Eqs. (8) and (9) can be applied directly to compute the reduced time and likewise the reduced time increment. If the temperature changes within a time increment, an approximation for small temperature increments is utilized,

20 $$ \Delta t_{\mathrm{red}} = \frac{ 2 }{A(T^{n+1}) + A(T^{n}) } \Delta t , $$

assuming one unique time–temperature shift function for all $R_{ij}(t)$ elements. Extension to individual shift functions is straightforward by modifying Eqs. (16) and (18). The time–temperature shift option is implemented for the viscoelastic behavior only, but not (yet) for the time-dependent thermal expansion relaxation/creep.

To control the accuracy of the solution, i.e. controlling $\Delta t$ in Eq. (16), an automatic time stepping procedure is implemented.

### Material parameters

For the measurement of relaxation data which can serve as input to a viscoelastic constitutive law there are standard procedures for simple loading scenarios. For intricate material response as given by $R_{ij}$, but in particular by $R_{12}$, a direct measurement can be very difficult or impossible. So, to calibrate the input from a series of (feasible) experiments some kind of optimization procedure might be useful.

In the present work the input data is generated by micromechanical type unit cell predictions which directly yields the viscoelastic relaxation matrix elements and thermal expansion relaxation coefficients. These functions of time are fitted by Prony series expansions.

## Verification and examples

For clarity of the following, the stress and strain components will be given with their full indication. For elements of the plane stress relaxation tensors (elastic and thermal expansion), the compact Voigt notation is maintained. Unless stated otherwise, all simulations are carried out at the WLF-reference temperature. All simulations employ four noded plane stress elements, using reduced integration for single element tests and full integration for the structural simulations. The element thickness is 1 mm.

### Isotropic material—single element tests

**Fig. 1:**
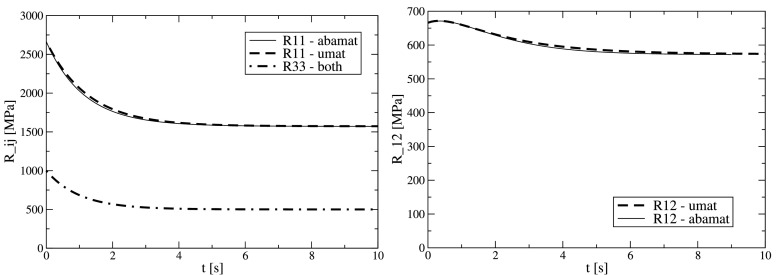
Single element predictions of the relaxation functions $R_{ij}(t)$ as response to Heaviside step strains by the ABAQUS built in material law (“abamat”) and the developed constitutive material law (“umat”) calibrated to the former one

Single element tests with isotropic linear viscoelastic material are carried out first. The element size is $1 \times 1~\mbox{mm}$. The ABAQUS built in material is used to obtain reference solutions, the generic material data (of some glassy polymer) is given in Table 1, which show shear relaxation only, plotted in Fig. 1 (left) as “R33”. Simulations are performed to compute the material parameters pertaining to the developed material model, i.e. the response to “uni-axial strain” loading is sought for. Here, uni-axial strain denotes the in-plane state, in the perpendicular direction plane stress is maintained throughout this work. A Heaviside step function is applied at $t=0$ and kept constant afterwards as $\varepsilon_{11}(t>0)=1$. The other in-plane strain components are $\varepsilon_{22}=0$ and $\gamma_{12}=0$ at any time. From this load case the $R_{i1}(t)$ components of the relaxation matrix can be deduced directly, see Fig. 1 (thin solid lines). Note that the response which contains the Poisson effect, $R_{12}(t) $, is non-monotonic. For completeness, the shear behavior $R_{33}(t)$ is added which exactly resembles the input, $G(t)$.

**Table 1:** Isotropic linear thermo-viscoelastic plane stress material data as input to the ABAQUS material law; instantaneous elastic moduli, bulk and shear relaxation by one Prony term each, WLF temperature shift data, and coefficient of thermal expansion

$\begin{array}{c@{\quad}c@{\quad}c@{\quad}c} E_{0}=2500~\mbox{MPa} & \nu _{0}=0.25 & (G_{0}=1000~\mbox{MPa}) \end{array}$
$\begin{array}{c@{\quad}c@{\quad}c@{\quad}c@{\quad}c} b=0 & (\tau ^{b}=1.0~\mbox{s}) & g=0.5 & \tau ^{g}=1.0~\mbox{s} \end{array}$
$\begin{array}{c@{\quad}c@{\quad}c@{\quad}c} T_{\mathrm{ref}}=0^{\circ }\,\mbox{C} & C_{1}=17 & C_{2}=50\end{array}$
$\begin{array}{c} \alpha _{0} = 1 \times 10^{-4} / ^{\circ }\,\mbox{C}\end{array}$

Based on this data, the material parameters for the UMAT are calibrated. The (sum of the) relative relaxation elements can be computed directly from the long term and short term response. The characteristic times can be deduced from the initial slope. If two Prony terms are required, a manual “trail and error” procedure is used.

The diagonal element $R_{11}(t)$ is well captured by one Prony term, for the off-diagonal element, $R_{21}(t)=R_{12}(t)$, at least two Prony terms are required to fit the non-monotonic behavior. The resulting material parameters for the UMAT are listed in Table 2. Now, these parameters and the developed UMAT are employed to simulate the same load case as before; see Fig. 1 (bold dashed lines). Comparison to the input shows some deviations but the resulting material behavior is considered sufficiently accurate. Note the difference in relaxation for various $R_{ij}$ term.

**Table 2:** Isotropic linear viscoelastic plane stress material data as input to the UMAT; instantaneous elasticity matrix elements, relaxation matrix elements by two Prony terms each, and WLF temperature shift data

$\begin{array}{@{}l@{\quad}c@{\quad}c@{\quad}c} R_{11 \; 0}=2666~\mbox{MPa} & R_{22 \; 0}=2666~\mbox{MPa} & R_{12 \; 0}=R_{21 \; 0}=666~\mbox{MPa} & R_{33 \; 0}=1000~\mbox{MPa}\\ \end{array}$
$\begin{array}[t]{c@{\quad}c@{\quad}c@{\quad}c@{\quad}c} k=1 & r_{11}=0.41 & r_{22}=0.41 & r_{12}=r_{21}=0.25 & r_{33}=0.25 \\ & \tau ^{r_{11}}=1.25\mbox{ s} & \tau ^{r_{22}}=1.25\mbox{ s} & \tau ^{r_{12}}=\tau ^{r_{21}}=2.0\mbox{ s} & \tau ^{r_{33}}=1.0\mbox{ s} \end{array}$
$\begin{array}{c@{\quad}c@{\quad}c@{\quad}c@{\quad}c} k=2 & r_{11}=0 & r_{22}=0 & r_{12}=r_{21}=-0.11 & r_{33}=0.25 \\ &\tau ^{r_{11}}=1.25\mbox{ s} & \tau ^{r_{22}}=1.25\mbox{ s} & \tau ^{r_{12}}=\tau ^{r_{21}}=0.6\mbox{ s} & \tau ^{r_{33}}=1.0\mbox{ s} \end{array}$
$\begin{array}{l@{\quad}c@{\quad}c} T_{\mathrm{ref}}=0 ^{\circ }\,\mbox{C} \quad C_{1}=17 \quad C_{2}=50\end{array}$

A series of additional tests and verifications are run based on single elements. Among them are classical relaxation test (under uni-axial stress), bi-axial loading, and arbitrary load cases. Besides the response to step functions, arbitrary ramp loading and load reversals are realized. All comparisons give satisfactory results. Also, the time–temperature shift function for temperature dependent viscoelastic material data is tested successfully as well as the automatic time stepping algorithm.

### Isotropic material—structural simulations

**Fig. 2:**
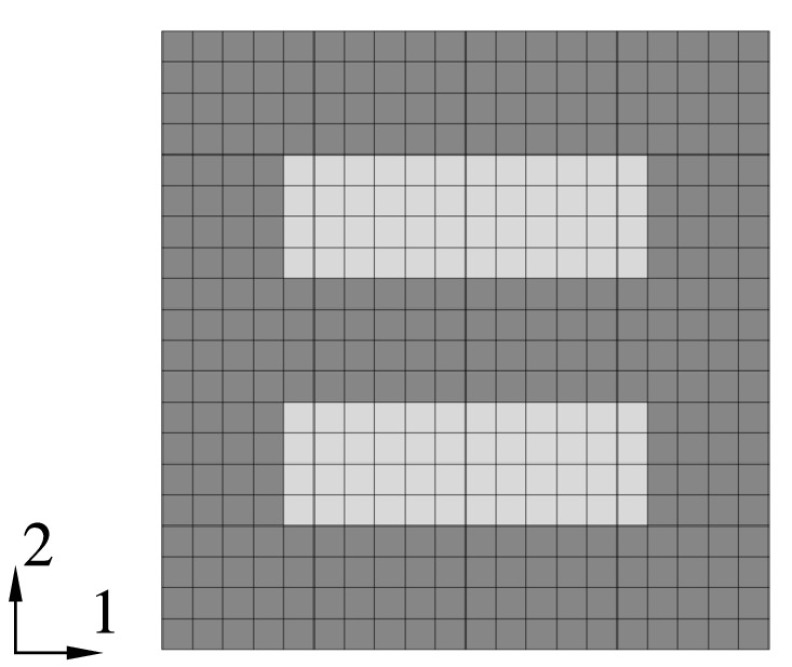
Structure with voids (light gray), also representing a unit cell of an orthotropic material

Next, simulations of structural problems with isotropic material are carried out which allow direct comparison with reference solutions. For this purpose a quadratic patch of unit length is discretized with $20 \times \mbox{20}$ plane stress elements. The patch contains two parallel rectangular voids with $0.2\times 0.6~\mbox{mm}$; see Fig. 2. The boundary conditions are “unit cell” like to be used later for homogenization; see Pettermann and Suresh (2000), Böhm (2004). The deformations of the nodes at the edges are coupled to the adjacent corner nodes of the model to remain on a straight line. The deformation of the model is controlled by the corner nodes, whereas the upper right corner is coupled to the others to maintain a parallelepipedic shape. This way, a unit cell type model is set up with three master nodes to apply loads and to read the response. Note that this does not give correct periodic boundary conditions (see e.g. Pettermann and Suresh 2000; Böhm 2004) under shear loads. However, it gives some homogenized behavior which sufficiently serves for the purpose of comparison in the present study.

The master nodes are used to apply a variety of loading scenarios as discussed above. The material model employed is either the ABAQUS built in one or the developed UMAT, whereas the voids are realized by setting the Young’s modulus to $E_{\mathrm{void}}=R_{33 \; 0} \times 10^{-3}$. The predicted master node responses and the local, time-dependent stress fields are compared, and for all studied load cases almost one to one agreement is found.

The implemented automatic time stepping as well as the consistent tangent matrix, i.e., the Jacobian, work correctly and the performance of the implemented material law is very satisfactory.

### Orthotropic porous material

**Fig. 3:**
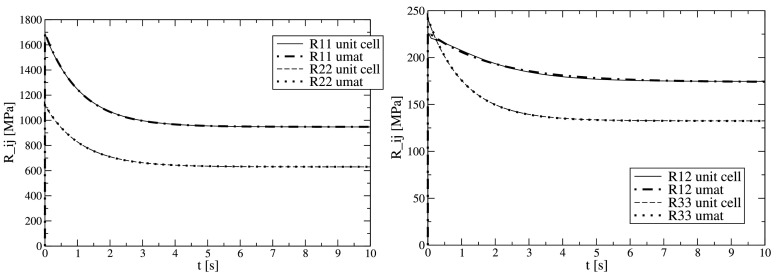
Orthotropic relaxation functions $R_{ij}(t)$ as response to Heaviside step strains; homogenization of a voided structure (“unit cell”) and single element predictions by the developed constitutive material law (“umat”) calibrated to the homogenized behavior

Now, the structure investigated in the previous section can be interpreted as porous material with orthotropic properties. By homogenization, the effective linear viscoelastic orthotropic plane stress behavior can be predicted. Applying the load cases of Heaviside step uni-axial strain in the two principal directions and for shear, the entire constitutive behavior can be predicted (as introduced in Sect. 5.1). The relaxation response is presented in Fig. 3 (denoted by “unit cell”), with $R_{12}(t)=R _{21}(t)$. All elements of $R_{ij}(t)$ are approximated by one Prony term, the material parameters are listed in Table 3. These material parameters together with the developed UMAT are now employed to run single element simulations. Comparison to the unit cell response is shown in Fig. 3 and excellent agreement can be seen for all elements. In addition to the direct comparison of the fitted response, a series of general load cases are investigated and compared successfully. The assumption of taking the same time–temperature shift as for the base material is confirmed to hold.

**Table 3:** Orthotropic linear viscoelastic plane stress material data as input to the UMAT; instantaneous elasticity matrix elements, relaxation matrix elements by one Prony term each, and WLF temperature shift data (taken to be equal to that of the input material)

$\begin{array}{c@{\quad}c@{\quad}c@{\quad}c} R_{11 \; 0}=1693~\mbox{MPa} & R_{22 \; 0}=1124~\mbox{MPa} & R_{12 \; 0}=R_{21 \; 0}=227~\mbox{MPa} & R_{33 \; 0}=242~\mbox{MPa} \end{array}$
$\begin{array}[t]{c@{\quad}c@{\quad}c@{\quad}c@{\quad}c} k=1 & r_{11}=0.440 & r_{22}=0.439 & r_{12}=r_{21}=0.234 & r_{33}=0.453 \\ & \tau ^{r_{11}}=1.09\mbox{ s} & \tau ^{r_{22}}=1.09\mbox{ s} & \tau ^{r_{12}}=\tau ^{r_{21}}=1.98\mbox{ s} & \tau ^{r_{33}}=1.08\mbox{ s}\end{array}$
$\begin{array}{c@{\quad}c@{\quad}c} T_{\mathrm{ref}}=0 ^{\circ }\,\mbox{C} & C_{1}=17 & C_{2}=50\end{array}$

Note the orthotropic behavior of the relaxation, too, which is fully captured by the present approach.

### Thermo-viscoelastic orthotropic composite

In this section an example is presented to study the thermal expansion relaxation. To this end the geometrical model of the structure from the previous examples is used, but the regions of the voids are now filled with rigid material to obtain a model composite. The reinforcement material is taken to be quasi-rigid with a Young’s modulus of $1 \times 10^{6}~\mbox{MPa}$, the Poisson ratio is set to 0.25. The isotropic coefficient of thermal expansion is set to $1 \times 10^{-6} / ^{ \circ }\,\mbox{C}$. For the matrix material the data from Table 1 is used, disregarding temperature dependence (i.e., no WLF shift is applied).

As in the previous examples, the unit cell is employed to perform material characterization, i.e., to compute the effective linear thermo-viscoelastic properties of the composite. The necessary (mechanical) load cases are simulated and the input data to the UMAT is fitted. Again, the off-diagonal term, $R_{12} (t)$, exhibits some curvature change around $t=2.0\mbox{ s}$ and is fitted by two Prony terms. The calibrated homogenized material data is listed in Table 4, graphical presentation is omitted.

**Table 4:** Orthotropic linear thermo-viscoelastic plane stress material data of a rigid inclusion reinforced model composite as input to the UMAT and the UEXPAN; instantaneous elasticity matrix elements, relaxation matrix elements, instantaneous coefficients of thermal expansion, and thermal expansion relaxation data

$\begin{array}{@{}l@{\quad}c@{\quad}c@{\quad}c} R_{11 \; 0}=5301~\mbox{MPa} & R_{22 \; 0}=3857~\mbox{MPa} & R_{12 \; 0}=R_{21 \; 0}=817~\mbox{MPa} & R_{33 \; 0}=714~\mbox{MPa}\end{array}$
$\begin{array}[t]{c@{\quad}c@{\quad}c@{\quad}c@{\quad}c} k=1 & r_{11}=0.419 & r_{22}=0.413 & r_{12}=r_{21}=0.203 & r_{33}=0.434 \\ & \tau ^{r_{11}}=1.20\mbox{ s} & \tau ^{r_{22}}=1.20\mbox{ s} & \tau ^{r_{12}}=\tau ^{r_{21}}=2.00\mbox{ s} & \tau ^{r_{33}}=1.00\mbox{ s} \end{array}$
$\begin{array}[t]{c@{\quad}c@{\quad}c@{\quad}c@{\quad}c} k=2 & r_{11}=0.000 & r_{22}=0.000 & r_{12}=r_{21}=-0.034 & r_{33}=0.000 \\ & \tau ^{r_{11}}=1.20\mbox{ s} & \tau ^{r_{22}}=1.20\mbox{ s} & \tau ^{r_{12}}=\tau ^{r_{21}}=0.35\mbox{ s} & \tau ^{r_{33}}=1.00\mbox{ s} \end{array}$
$\begin{array}{c@{\quad}c} \alpha _{1 \; 0}=5.16\times 10^{-5}/^{\circ }\,\mbox{C} & \alpha _{2 \; 0}=7.58\times 10^{-5}/^{\circ }\,\mbox{C}\end{array}$
$\begin{array}{c@{\quad}c@{\quad}c} k=1 & a_{1}=-0.019 & a_{2}=-0.045 \\ & \tau ^{a_{1}}=1.00\mbox{ s} & \tau ^{a_{2}}=1.30\mbox{ s}\end{array}$

**Fig. 4:**
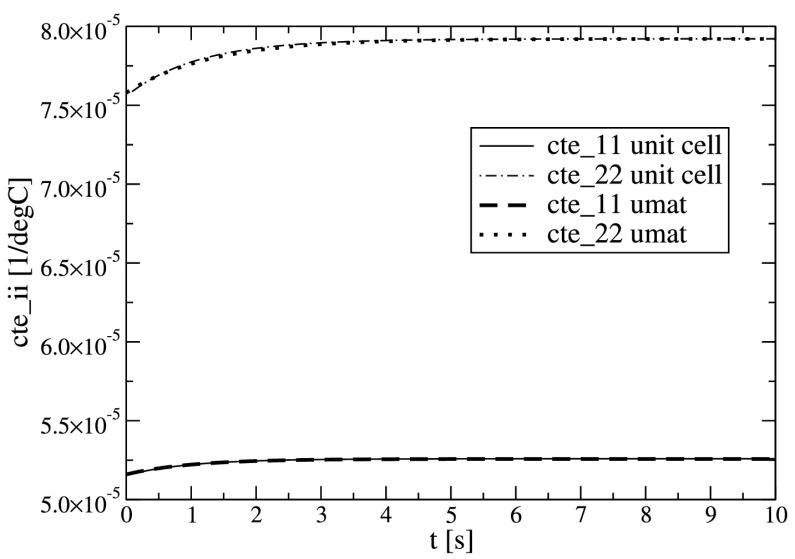
Orthotropic thermal expansion relaxation/creep functions $\alpha_{i}(t)$ as response to unit temperature Heaviside step; homogenization of a rigid inclusion reinforced model composite (“unit cell”), and single element predictions by the developed thermal expansion law (“uexpan”) calibrated to the homogenized behavior

In addition, the effective thermal expansion behavior of the composite can be predicted by the unit cell simulations. A Heaviside temperature step, $\vartheta (t>0)=1$, from an undeformed, stress free configuration is applied and the thermal expansion response is evaluated. The constituents of the model composite posses different coefficients of thermal expansion, thus, a uniform temperature change of the composite induces stresses and strains in the constituents (the stresses, of course, are self-equilibrated). Since these stresses and strains show relaxation and creep, the effective thermal expansion shows relaxation/creep, too. Note that no thermal diffusion is involved here. Due to the orthotropic material symmetry there are two independent thermal expansion relaxation/creep functions. The corresponding unit cell predictions are presented in Fig. 4 (thin lines).

To obtain input data to the UEXPAN subroutine, the thermal expansion response is fitted, and the material data is listed in Table 4. It comprises the two instantaneous coefficients of thermal expansion and the corresponding relaxation/creep functions with one Prony term each. The fitted behavior is presented in Fig. 4 (thick dashed lines) for comparison with the unit cell predictions.

So far, the material data input is calibrated and other thermo–viscoelastic load cases are studied. Temperature changes under fully and uni-axial in-plane constraint are simulated which give rise to thermally induced stresses. For both cases excellent agreement is found for the unit cell response and the UMAT/UEXPAN prediction. For comparison, the same load cases are simulated with coefficients of thermal expansion taken to be time independent, i.e. suppressing thermal expansion relaxation. As expected, the stress responses deviate for increasing time.

## Summary

A constitutive material law for linear thermo-viscoelasticity in the time domain with full anisotropy is presented. For the linear viscoelastic material behavior the anisotropic material symmetry is not only considered for the elastic part. Also for the relaxation response, full anisotropy is realized with the appropriate number of independent material values. The formulation is based on a time-dependent elasticity tensor for which each element possesses its own relaxation function. This way, the mutual coupling of the normal components, i.e., Poisson type effects, is accounted for. The relaxation function for each tensor element is prescribed by its individual Prony series expansions.

An incremental algorithm is set up which gives the time-dependent stress response to a prescribed incremental strain history. State dependent internal variables are utilized to account for the loading history, and the consistent material Jacobian is formulated. Temperature dependence is realized by a time–temperature shift function of the Williams–Landel–Ferry type. It is implemented for orthotropic material symmetry under plane stress assumption into a FEM package. The extension to tri-axial stress and strain states and to general anisotropy is straightforward.

The thermal expansion relaxation/creep is considered as inherent material behavior. It is treated in analogy to the mechanical, i.e., viscoelastic, model. The material behavior is described by instantaneous coefficients of thermal expansion and their individual relaxation functions. The time-dependent thermal strain is modeled as a function of the applied temperature change. Again, internal state variables are used to account for the temperature history.

Various tests on isotropic and orthotropic plane stress problems are carried out successfully for verification and validation. The performance is found to be very satisfactory. Orthotropic test cases are set up by considering heterogeneous model composites and employing a periodic unit cell type approach. Thereof, homogenized material data is computed, serving as input to the developed material law. Also the thermal expansion relaxation behavior is investigated.

Finally, the developed constitutive laws provide tools for structural analyses of components made from orthotropic linear thermo–viscoelastic materials.
